# Growth, structure, and morphology of van der Waals epitaxy Cr_1+δ_Te_2_ films

**DOI:** 10.1186/s11671-023-03791-y

**Published:** 2023-02-24

**Authors:** Xiaodan Wang, Hua Zhou, Lihui Bai, Hui-Qiong Wang

**Affiliations:** 1 Engineering Research Center of Micro-Nano Optoelectronic Materials and Devices, Ministry of Education; Fujian Key Laboratory of Semiconductor Materials and Applications, CI Center for OSED, and Department of Physics, Xiamen University, Xiamen, 361005 People’s Republic of China; 2 School of Physics, Shandong University, Jinan, 250100 People’s Republic of China

**Keywords:** Cr_1+*δ*_Te_2_, Scanning tunneling microscopy, Film growth, Surface structure, Morphology evolution, Mica

## Abstract

The preparation of two-dimensional magnetic materials is a key process to their applications and the study of their structure and morphology plays an important role in the growth of high-quality thin films. Here, the growth, structure, and morphology of Cr_1+*δ*_Te_2_ films grown by molecular beam epitaxy on mica with variations of Te/Cr flux ratio, growth temperature, and film thickness have been systematically investigated by scanning tunneling microscopy, reflection high-energy electron diffraction, scanning electron microscope, and X-ray photoelectron spectroscopy. We find that a structural change from multiple phases to a single phase occurs with the increase in growth temperature, irrespective of the Cr/Te flux ratios, which is attributed to the desorption difference of Te atoms at different temperatures, and that the surface morphology of the films grown at relatively high growth temperatures (≥ 300 °C) exhibits a quasi-hexagonal mesh-like structure, which consists of nano-islands with bending surface induced by the screw dislocations, as well as that the films would undergo a growth-mode change from 2D at the initial stage in a small film thickness (2 nm) to 3D at the later stage in thick thicknesses (12 nm and 24 nm). This work provides a general model for the study of pseudo-layered materials grown on flexible layered substrates.

**Supplementary Information:**

The online version contains supplementary material available at 10.1186/s11671-023-03791-y.

## Introduction

In recent years, 2D magnetic materials have aroused significant attention for they are crucial to the development of practical spin electric devices to satisfy the increasing requirement of high-efficiency and small-volume electron devices [1, 2]. Transition-metal dichalcogenides (TMDs) are one of the promising classes of materials in this field [3]. A typical example is the chromium telluride compounds [Cr_1+*δ*_Te_2_ (0 < *δ* ≤ 1)], many members (CrTe, Cr_3_Te_4_, Cr_2_Te_3_, Cr_5_Te_8_, CrTe_2_, et al.) of which have been demonstrated to exhibit a variety of properties favorable for applications such as room-temperature intrinsic ferromagnetism [4, 5], perpendicular magnetic anisotropy [6, 7], air-stability [8, 9], and half-metallicity [10, 11]. Beyond these outstanding properties, anomalous Hall effect (AHE) and topological Hall effect (THE) have also been observed in the Cr_1+*δ*_Te_2_ family. In 2018, Zhao et al. reported an AHE noted as direct experimental observation of THE in CrTe films [12]. In 2019, Wang et al. reported a THE attributed to the chiral effect derived from noncoplanar spin configurations in Cr_5_Te_8_ [13]_._ In 2020, Zhou et al. revealed a method to generate THE by embedding Bi nanosheets into bulk Cr_2_Te_3,_ giving a magnetic skyrmion scenario [14]. In 2021, Huang et al. reported a pronounced room-temperature THE in Cr_1.2_Te_2_ and facilitated the realization of skyrmion-based devices [15]. In 2022, Tang et al. observed colossal AHE in Cr_5_Te_8_ [16]. More interestingly, the layered structure of chromium telluride compounds [17, 18] makes this material possible to be well compatible with most 2D van der Waals materials including topological and Weyl semimetal materials. Researchers have designed many new interfaces based on Cr_1+*δ*_Te_2_ to explore the modern advanced physical phenomenon and have gained many achievements. For example, Zhang et al. observed THE signals in CrTe_2_/Bi_2_Te_3_ heterostructures and proposed that chiral spin texture exits at the interface [19]. Despite the tremendous progress has been made on Cr_1+*δ*_Te_2_, there are still many challenges in this field for practical use like growing high-quality samples [20].

On the other hand, with the rapid development of electronic information technology and nanotechnology in modern society, a new electronic technology developed by using flexible or scalable devices and their integrated systems has emerged: flexible electronics technology [21]. The devices based on this technique can realize data collection, processing, and transmission, and can display just like traditional rigid electronic devices. The applications of this technique are wide-ranging such as health monitoring [22], data-feedback training [23], and human–computer interaction [24]. In recent years, numerous studies based on this technology have attracted great interest such as the design and fabrication of flexible, portable, and wearable devices [25–27] to the increasing demands of the internet of things. Among them, flexible electronics integrating spintronics is a promising applied prospect in the areas of lightweight and flexible personal electronics [28]. At present, the fabrications of flexible magnetic devices are realized mainly by transferring the 2D van der Waals magnetic materials obtained by the mechanical exfoliation method to the flexible substrates or combining the 2D van der Waals magnetic materials with other 2D van der Waals electronic materials. However, the method of mechanical exfoliation is difficult to provide a clean interface. Preparing ultra-thin films on flexible substrates by direct growth methods may be an effective way to address this issue. Pseudo-layered Cr_1+*δ*_Te_2_ thin film is one of the major material candidates for spintronics devices which can be fabricated using bottom-up growth methods such as molecular beam epitaxy (MBE) [17], avoiding the shortcomings such as process uncontrollability, size uncontrollability, and interface undesirability, induced by mechanical exfoliation method. Moreover, non-vdW Cr_1+*δ*_Te_2_ has been proven to be able to be combined with vdW material in vdW epitaxy growth mode [17]. Excitingly, van der Waals epitaxy can be applied to a wide range of materials and provide a large degree of freedom in preparing heterostructures by combing different materials [29]. It occurs when the interaction between the epitaxial layer and the substrate material is weak van der Waals interactions but not strong chemical bonds. The major advantageous consequence compared to conventional epitaxy is that van der Waals epitaxy makes it possible to grow high-quality epitaxial material without considering the demand for lattice matching between the film and the substrate [30, 31]. Mica is a layered material with interlayer van der Waals interactions that can be peeled off into thin layers with few atomic layers [28] and exhibits well-transparent and mechanically flexible properties which made it an alternative substrate in the transparent flexible electrodes [32, 33]. Furthermore, vdW epitaxy on mica has been demonstrated to provide a basic arena for developing multifunctional flexible X-tronics [31]. Thus, integrating Cr_1+*δ*_Te_2_ thin films on flexible vdW mica substrates [34] is perhaps a fascinating strategy to accelerate the practical application of Cr_1+*δ*_Te_2_ materials on flexible electronics.

In this work, we have grown Cr_1+*δ*_Te_2_ films on flexible mica substrates with variations of Te/Cr flux ratio, growth temperature, and film thickness, as well as systematically investigated the growth conditions and surface structures of the films. The growth conditions of the films are explored using X-ray diffraction (XRD), X-ray photoelectron spectroscopy (XPS), and scanning electron microscopy (SEM). We find that multiple phases are present in the growth process at a lower growth temperature (200 °C) and a single phase is formed at higher growth temperatures (300 °C, 360 °C, 460 °C), irrespective of the Te/Cr flux ratios. The surface structures are investigated by scanning tunneling microscopy (STM). It is found that the surface morphology is affected by the growth temperature and film thickness, and the screw dislocations and grain boundaries emerge during the growth in high growth temperatures. And the morphology evolution hints that the film growth mode initially is 2D, then a transition growth mode between 2 and 3D, and last is 3D island with the increase in the film thickness. Our work paves the way for further research on the fundamental physics of vdW heterostructures formed by non-vdW materials and flexible layered substrates and sets a foundation for its application in flexible spintronics.

## Experimental section

### Growth

The samples were grown on mica substrates by co-evaporation of chromium and tellurium with a high purity of 99.999% (Aladdin, America) from two Knudsen cells in an ultra-high vacuum molecular beam epitaxy (MBE, Createc. Germany) chamber with the base pressure and growth pressure in the growth chamber maintained below 1 × 10^−10^ mbar and 1 × 10^−8^ mbar, respectively. The flexible mica substrates were freshly cleaved from smooth fluorphlogopite mica sheets (KMg_3_(AlSi_3_O10)F_2_, purchased from Taiyuan Fluorphlogopite Co. Ltd., Changchun City, China) in air and pasted with two electrodes made by dripping suitable silver paste on them and were baked at 300 °C for 30 min to solidify and degas the silver paste before being transferred to the load-lock chamber. Subsequently, the substrates were outgassed in a vacuum with 460 °C for at least 30 min before growth with the pressure maintained below 1 × 10^–8^ mbar, and then, the substrates were transferred to the MBE chamber. The Te- and Cr-deposition rates were calibrated with a quartz microbalance and the Te/Cr flux ratios were set to 100 and 130 for the low- and high-Te/Ce flux ratio, respectively, as discussed below. The substrate temperatures during growth were set to 200 °C, 300 °C, 360 °C, and 460 °C.

### Characterization

The substrate after the annealing treatment was measured in situ using reflection high-energy electron diffraction (RHEED) in the MBE chamber equipped with an electron gun generating a 12 keV electron beam, and the RHEED image from the mica substrate after annealing treatment shown in Additional file 1: Figure A1 in which the red arrows label the bright Kikuchi lines, indicates that the substrate surface is quite smooth without contamination from the silver paste after the annealing treatment. The samples after the growth were transferred in situ from the MBE chamber to the STM chamber through the preparation chamber. The STM experiments were performed with a Createc low-temperature scanning tunneling microscopy (LT-STM) with a mechanically cut Pt/Ir tip with a diameter of 0.2 mm, operating at approximately 77 K in an ultra-high vacuum (UHV) chamber at a pressure of 1 × 10^–10^ mbar. And the corresponding scanning current and voltage were 0.1 nA and 3–8 V, respectively. XRD measurements were carried out by an x-ray diffractometer (SmartLab 3KW, Japan) that has a Cu Kα source with a wavelength *λ* = 1.54 Å. The step size and the speed for the XRD *θ-2θ* scans over the range 10° < 2*θ* < 90° were 0.02° and 3° min^−1^, respectively. XPS (Thermo Fisher ESCALAB Xi+) was used to characterize the change in the chemical composition. The X-ray tube with Al kα-radiation (1486.6 eV) was used as a source of ionizing radiation and the X-ray photon energy was calibrated by the Ag 3*d*_5/2_ XPS peak with a binding energy of 368.2 eV [35]. The surface morphology of the films was also observed by scanning electron microscope (SEM, Thermo Scientific, USA) and atomic force microscope ((AFM, SPM-9700HT, Shimadzu Co.). Film thickness was estimated by the cross section transmission electron microscope (TEM) result shown in Additional file 2: Figure A2.

## Results and discussion

### Crystal structures

**Fig. 1:**
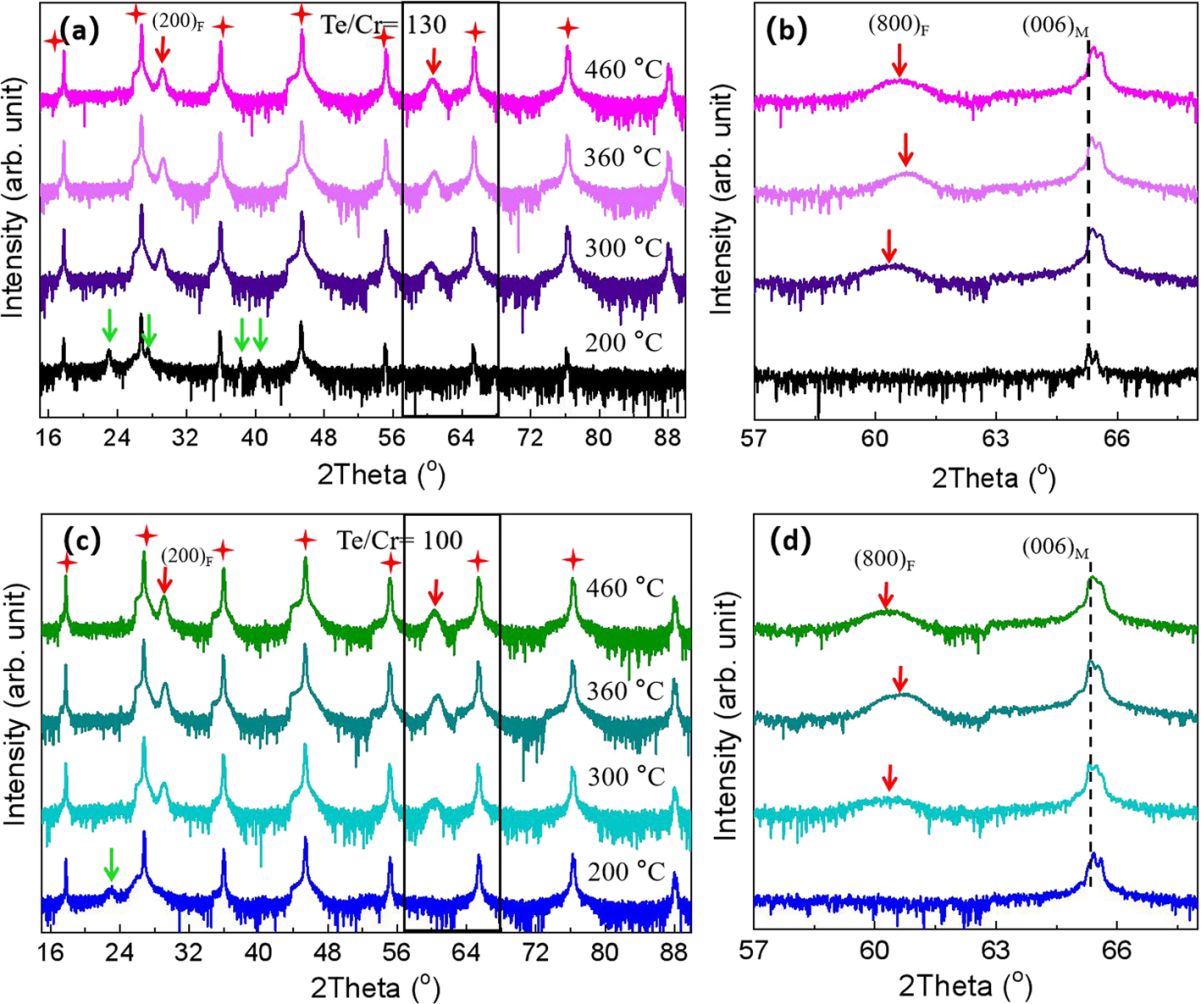
XRD results of the films grown at 200 °C, 300 °C, 360 °C, and 460 °C. **a**, **c** XRD results from the series of samples with Te/Cr flux ratios of 130 and 100, respectively; **b**, **d** corresponding to the amplification of the peak areas marked by the black rectangle in **a**, **c**, respectively. Here, the red stars label the peaks from the mica substrate, the red arrows label the peaks from the Cr_1+*δ*_Te_2_ films having different phases varying by the value of *δ* but almost identical structure without taking into account the intercalation of Te atoms, and the green arrows label the peaks from some other phases outside the Cr_1+*δ*_Te_2_ family, respectively

First, we focus on the film structures and orientations depending on the Te/Cr flux ratio and growth temperature. Figure 1 shows the XRD images from the two series of samples with the Te/Cr flux ratios of 130 and 100, respectively, and the growth temperature (*T*_g_) of 200–460 °C. The XRD 2*θ*/*θ* scan profiles of the films grown at various growth temperatures (*T*_g_ = 200 °C, 300 °C, 360 °C, and 460 °C) with a fixed flux ratio of Te/Cr = 130 are shown in Fig. 1a. The peaks indicated by the red stars are assigned to the diffraction from the mica substrate. Several weak peaks at 22.93°, 27.64°, 38.28°, and 39.83°, which are close to the 2*θ* value expected from the planes of Cr_5_Te_6_-$(1\overline{1 }\overline{1 })$(CIF: mp-1226086), CrTe_3_-$(\overline{1 }23)$(CIF: mp-540922), Cr_5_Te_6_-(300) (CIF: mp-1226086) and CrTe_2_(10$\overline{1 }$2) (CIF: mp-685055), appear in the sample grown at 200 °C. The peaks at 29.2° and 60.6° labeled by the red arrows appear in the films grown at higher temperatures of *T*_g_ = 300 °C, 360 °C, and 460 °C, which are expected from the (200) and (800) planes of Cr_3_Te_4_, a member with the *δ* value of 0.5 being chosen as a representative of the Cr_1+*δ*_Te_2_ family to label the diffraction planes. This result indicates that the films grown at relatively low temperatures are apt to form multiple-phase structures. Figure 1b corresponds to the amplified image of the area labeled by the black rectangle in Fig. 1a. It is seen that slight position shifts are introduced to the peak position of (800)_F_ (F represents the film) with the peak position first shifted toward higher degrees and then shifted toward lower degrees as an increase of the growth temperature in Fig. 1b. This result is deemed to stem from the competition between lattice relaxation and desorption of Te atoms. In the range of growth temperature from 300 to 360 °C, the desorption efficiency of Te atoms is increased rapidly and leads to a slight decrease in the lattice constant. As a consequence, the peak position is slightly shifted toward higher degrees in XRD images. When the growth temperature is larger than 360 °C, the effect of the lattice-relaxation processes will become profound. Because the in-plane lattice constants (*a* = 0.4 nm; *b* = 0.69 nm) of the film [36] are smaller than that of the substrate (*a* = 0.53 nm; *b* = 0.93 nm) [37, 38], the lattice relaxation induces the increase of the out-plane lattice constant, leading a slight shift toward the lower degree, which can explain the shifts of the peak position in the XRD results illustrated by the red arrows in Fig. 1b. Figure 1c, d shows the XRD results of the sample grown at various growth temperature (*T*_g_ = 200 °C, 300 °C, 360 °C, and 460 °C) with a lower fixed flux ratio of Te/Cr = 100. The peaks labeled by the red stars are from the mica substrate. It can be seen from the blue curve line corresponding to the sample grown at 200 °C in Fig. 1c that only one peak located at 22.93° appears, which means that some structure phases are reduced by decreasing the Te/Cr flux ratio comparing with a higher Te/Cr flux ratio of 130. As for the samples with *T*_g_ = 300 °C, 360 °C, and 460 °C, the XRD results shown in Fig. 1c, d are very similar to that of the samples with a higher Te/Cr flux ratio of 130. We also carried out the corresponding XRD characterizations for the samples grown at various growth temperatures (*T*_g_ = 200 °C, 300 °C, 360 °C, 460 °C) with a further lower fixed flux ratio of Te/Cr = 35, and the details are shown in Additional file 3: Figure A3. As can be seen, the multiple-phase structures disappear in the 200 °C sample, instead, the peaks at 29.2° and 60.6° (labeled by the red arrows) appeared, which means that further reduction in Te/Cr flux ratio to 35 completely eliminated the multiple phases, and made the phase structure consistent with higher-growth-temperature cases. Likewise, for the 300 °C, 360 °C, and 460 °C samples, the XRD results are very similar to that of the samples with higher Te/Cr flux ratios of 100 and 130. The results reveal that the Te/Cr flux ratio mainly influences the structure phase of the film grown at lower temperature regions (≤ 200 °C) and scarcely affects the film structures with higher growth temperatures (≥ 300 °C).

### Chemical properties

**Fig. 2:**
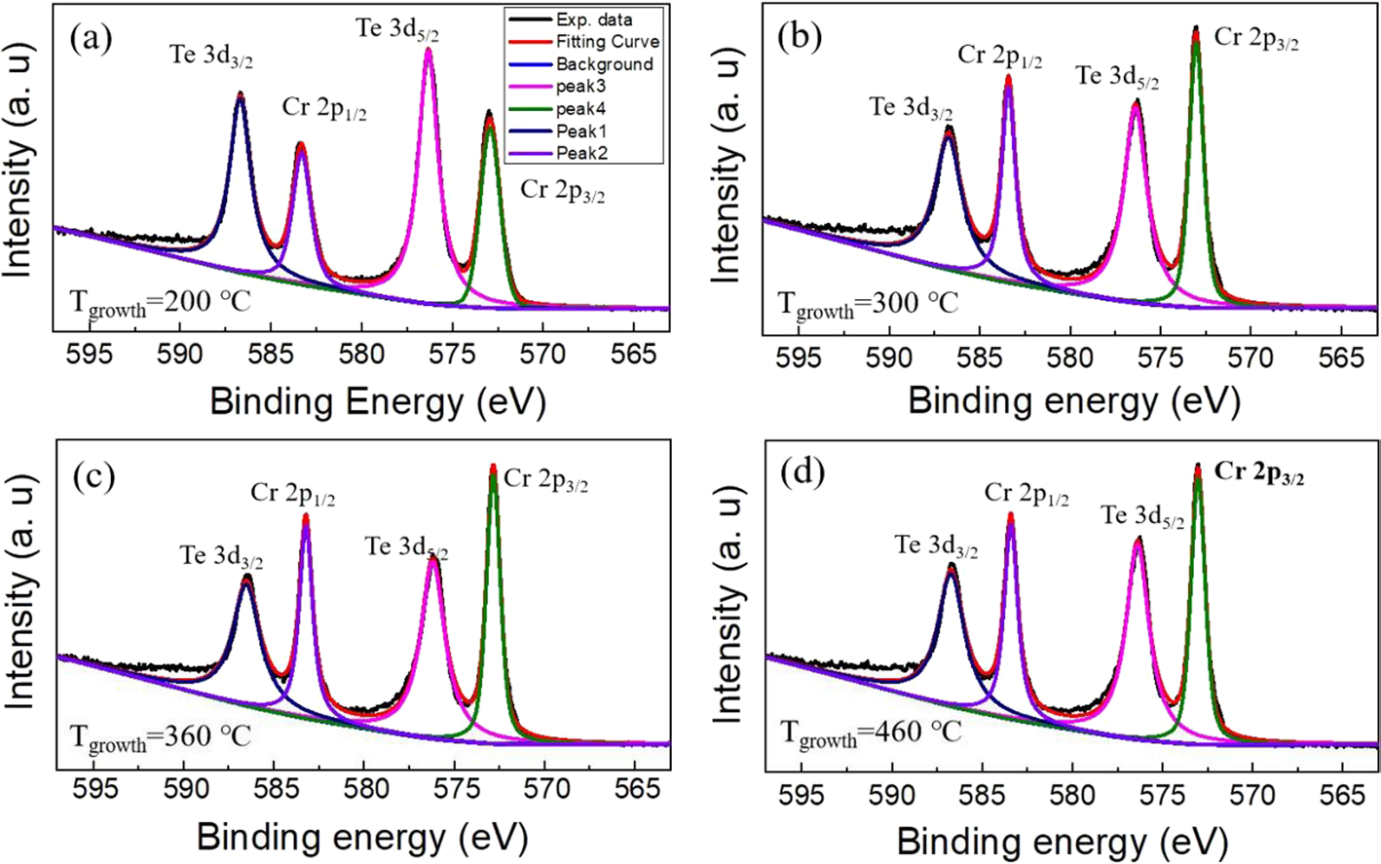
XPS spectra. High-resolution Cr 2*p* and Te 3*d* core level spectra of films with the Te/Cr flux ratio of 100 grown at **a** 200 °C, **b** 300 °C, **c** 360 °C and **d** 460 °C

XPS measurements were carried out to study the change in the chemical composition of the as-grown samples. Figure 2a–d shows the Cr 2*p* and Te 3*d* core level XPS spectra of the films with the Te/Cr flux ratio of 100 grown at various growth temperatures (*T*_g_ = 200 °C, 300 °C, 360 °C, and 460 °C). The spectra were deconvolved by fitting a pure chromium spectrum to the composite peak envelope to determine the contributions of different components in the samples. The peaks located at 573 eV and 583 eV are assigned to Te 3*d*_5/2_ + Cr 2*p*_3/2_ and Te 3*d*_3/2_ + Cr 2*p*_1/2_, respectively, since the binding energies of the Cr 2*p* and Te 3*d* core levels are very close to each other and it is hard to separate them out [39–41]. The peaks located at about 576 eV and 587 eV are related to Te 3*d*_3/2_ and Te 3*d*_5/2_, respectively, which are attributed to the formation of Te_*x*_O_*y*_ [40, 42], since the samples are exposed to air for some time before the XPS measurements. The spectra were fitted by two-component peaks, i.e., Cr 2*p*_3/2_ + Te 3*d*_5/2_ (Cr 2*p*_1/2_ + Te 3*d*_3/2_), and Te from Te_*x*_O_*y*_, for the lower (higher) binding energy peaks. The stoichiometric ratio between Te and Cr elements according to the XPS data is also briefly discussed in the Supplementary Information (see Additional file 5: Table A1 and the corresponding discussion), showing that the atomic ratio of Te/Cr with *T*_g_ of 200 ℃, 300 ℃, 360 ℃, and 460 ℃ is approximately 0.8, 0.6, 0.6, and 0.6, respectively. Remarkably, it is hard to determine the practical chemical formula stoichiometry of the sample only relying on the XPS results, since numerous Te atoms in the surface may be oxidized to be Te_*x*_O_*y*_, forming a layer in the surface. And it should be noted that the XPS technique for element-composition analysis is normally furnishing semi-quantitative results (within 10–20%) [43]. Therefore, it is hard to obtain the exact value of Cr and Te element concentration in the film according to the above-mentioned two factors. That is to say, it is difficult to determine the value of δ in the Cr_1+δ_Te_2_ family on basis of the XPS analysis results.

### Morphology

**Fig. 3:**
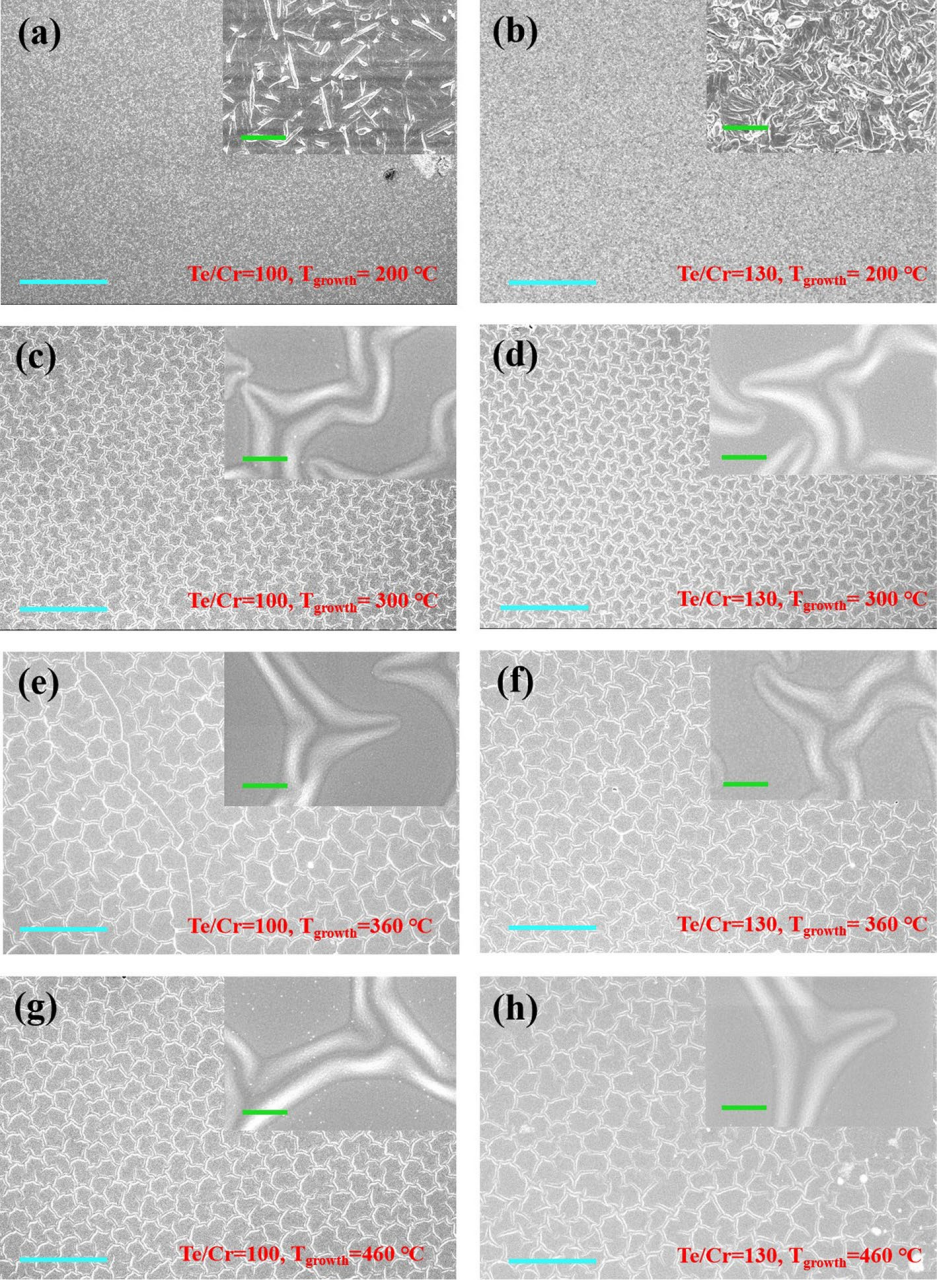
SEM images. SEM images of the films with the Te/Cr flux ratio of 100 (left column) and 130 (right column) grown at **a**, **b** 200 °C, **c**, **d** 300 °C, **e**, **f** 360 °C, and **g**, **h** 460 °C. The insets show the corresponding magnified SEM images. Scale bars are 20 μm and 1 μm (inset), respectively

Figure 3 shows the SEM images of the films with the flux ratio of Te/Cr = 100 (left column) and Te/Cr = 130 (right column). And the corresponding growth temperatures are 200 °C (*a*–*b*), 300 °C (*c*–*d*), 360 °C (*e*–*f*), and 460 °C (*g*–*h*), respectively. The insets show the corresponding zoom-in SEM mages. The SEM images of the lower growth temperature samples (*T*_g_ = 200 °C) in Fig. 3a, b suggest a uniform surface morphology, consisting of random arrangement nanowires (the lower flux ratio, Te/Cr = 100) and nanosheet (the higher flux ratio, Te/Cr = 130) respectively, as shown in the zoom-in SEM images (corresponding inset). Interestingly, when the growth temperature is elevated larger than 300 °C, a kind of irregularly hexagonal mesh structure appears in the film surface via self-assembly mode (Fig. 3c–h). And the density (or size) of the hexagonal mesh structure becomes smaller (or larger) with the increase in growth temperature. This result is well consistent with the AFM result (see Additional file 4: Figure A4a-c). Besides, a comparison of the AFM images reveals root-mean-square (RMS) values of 51.23, 59.27, and 63.36 nm for 300 °C, 360 °C, and 460 °C samples, respectively, showing an upward trend as the increase of growth temperature. Actually, the edge touchline connecting the structure mesh is broken in the middle part, as shown in the zoom-in SEM images in the insets of Fig. 3c–h. To the best of our knowledge, such kind of morphology feature has not been reported in previous references on the Cr_1+*δ*_Te_2_ film. We believe these swelling mesh structures are probably from the evolution of grain boundaries, a large amount of which are found in the in situ STM images (discussed below), showing a surface morphology evolution of the grain boundary into mesh structure on the film surface with the increase in the film thickness.

### Surface structure and evolution

**Fig. 4:**
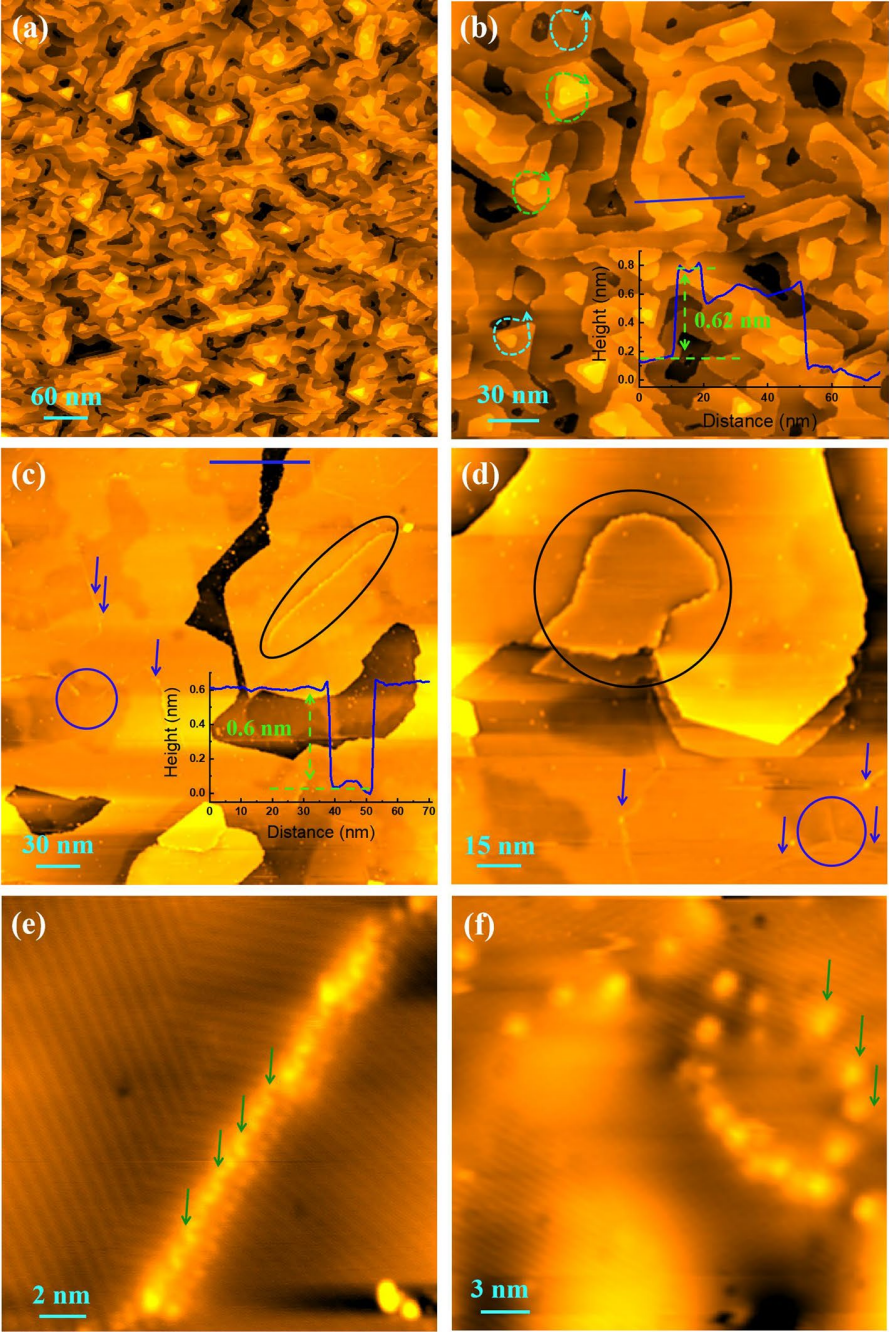
STM images. **a**, **b** The 24 nm thickness sample growth at 360 °C with the Te/Cr flux ratio of 100. Here, the green and light blue dashed circles in **b** label the clockwise and anticlockwise spirals. **c**, **d** The 8 nm thickness sample grown at 360 °C with a Te/Cr flux ratio of 100. **e**, **f** Enlarged STM images from the 8 nm sample show two kinds of boundaries, as labeled by the green arrows

To investigate the surface structures of the mesh, in situ STM was employed for the Cr_1+*δ*_Te_2_ films grown at 460 °C. Figure 4a is a large-area STM image from the 24 nm sample, showing that the film surface consists of many islands with bending terraces, and there exist many spiral islands in the film. In the higher-resolution STM image shown in Fig. 4b, the spiral terraces can be more clearly seen and it shows two types of rotations with one rotating clockwise and the other anticlockwise as labeled by the green and red dashed circles respectively. This spiral growth mode is common for some other 2D van der Waals materials and semiconductor films [44, 45] and such spiral growth mode has also been observed previously in Cr_2_Te_3_ films grown on Si(111) substrates [46]. And the formation of this spiral growth is thought to be associated with screw dislocations according to pioneering theoretical research by Burton, Cabrera, and Frank (BCF) [47], which provides hints that there are many screw dislocations in the film surfaces. The STM images of the 8 nm sample are also presented in Fig. 4c–f. The large-scale images are shown in Fig. 4c, d, indicating a rather flat surface of the film with many grain boundaries, as labeled by the blue arrows. As can be noticed, there emerge several inter-sectional parts of three boundaries at some localized regions, as marked by the blue circles. This feature can be associated with the mesh-structure feature of SEM images (Fig. 3c–h), and hence, the evolution of the grain boundaries may be considered as the origin of the forming of the mesh structures. And the boundaries should be the nucleation of the screw dislocations at the subsequent layer since the formation energy at the boundary is generally lower [48, 49]. The small-scale images (Fig. 4e, f) show two types of gain boundaries, with one (straight, Fig. 4e) consisting of humping atoms arranged continuously and straightly, and the other (bending, Fig. 4f) consisting of atoms arranged homogeneously but discontinuously and nonlinearly, as labeled by the green arrows. Notably, the parallel stripe structure presented in the surface (Fig. 4e) reveals a 2 × 1 periodicity, which is a feature of the STM images of Cr_3_Te_4_ film [41]. Moreover, the profiles (plotted along the blue lines) of the insets in Fig. 4b, d show that the step height is about 0.6 nm, which is comparable to the lattice constant of Cr_3_Te_4._ (0.62 nm [36]). Therefore, the films are speculated to be Cr_3_Te_4_.

**Fig. 5:**
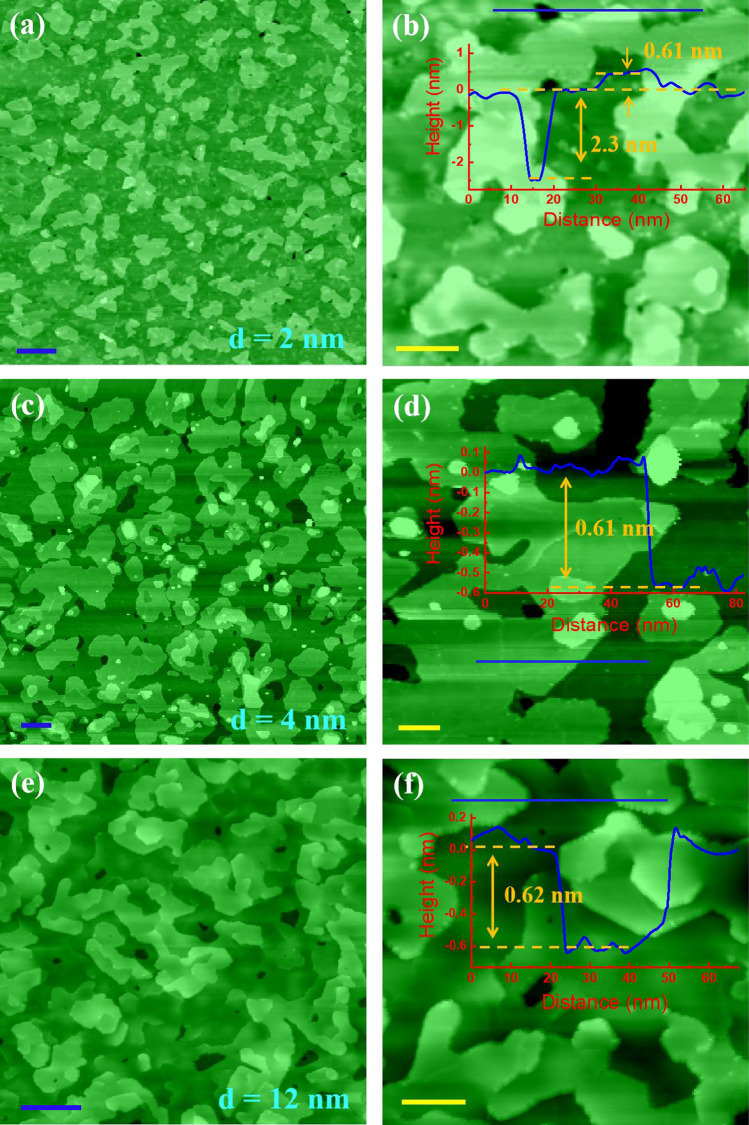
Surface structure evolution of the films. **a**, **c**, **e** STM images (*V* = 8 V, *I* = 0.1 nA) from the films with thicknesses of 2 nm, 4 nm, and 12 nm, respectively. **b**, **d**, **f** Zoomed-in STM images (*V* = 6 V, *I* = 0.1 nA) of **a**, **c**, **e**, respectively. The insets in **b**, **d**, **f** plot the profiles along the blue lines from left to right. The blue and yellow scale bars represent 50 nm and 20 nm, respectively

In situ STM observations of the films grown at 360 °C with a Te/Cr flux ratio of 100 and five different kinds of thickness [2 nm, 4 nm, 8 nm, 12 nm, and 24 nm], shown in Figs. 4a–d and 5a–f, reveal the surface structure evolution. Figure 5 corresponds to the thicknesses of 2 nm, 4 nm, and 12 nm. And the images in the right column are the higher-resolution ones of the corresponding images in the left column, and the profiles of the insets in the right column are plotted along the blue lines. Apparently, there exist many 2D islands with no dislocations or twist grain boundaries induced by lattice mismatch in the 2 nm sample, as shown in Fig. 5a, reflecting a vdW epitaxy mode [17]. And the island height is measured to be 0.6 nm, approximately equaling the lattice constant along the c-axis (0.62 nm [36]) of the Cr_3_Te_4_, as labeled by the black arrows in the inset of Fig. 5b, indicating a 2D growth mode. While for the 4 nm sample, the islands tend to coalesce and a lot of holes emerge, as shown in Fig. 5c. At this stage, there emerges a small number of spirals, meaning that the density of the screw dislocation on the surface is low. The height of the merged islands is measured to be 0.63 nm, undergoing little change compared with the case of the 2 nm sample, as marked by the red double arrow shown in Fig. 5d. This result implies that the growth from 2 to 4 nm experienced a formation-and-coalescence process of 2D islands and the film did not exhibit complete two-dimensional growth behavior in this growth stage. Remarkably, in the case that the film thickness is very small (2 nm), there are a few holes among the islands on the surface. The hole depth is determined to be 2.3 nm from the STM image, as labeled by the red double arrow in the inset of Fig. 5b. Remarkably, this value is well consistent with the result estimated by TEM results, meaning that maybe the hole depth here could be used to estimate the film thickness, and therefore, the film thickness of the 2 nm sample is determined to be 2.3 nm. While on the 8 nm sample surface (Fig. 4c–f), the 2D islands, boundaries, and screw dislocations emerge gradually. When the film thickness gradually increases to approximately 12 nm, the formation of 3D islands on the surface is obviously seen, as shown in Fig. 5e. It is shown in the inset of Fig. 5f that the island height is a few times the length of the lattice constant c (~ 0.6 nm). And some spirals indicating the emergence of screw dislocations on the island terraces can also be seen in Fig. 5f, as labeled by the violet circles. When the film thickness further increases and reaches approximately 24 nm, the film surface becomes rougher and the density of the screw dislocation becomes larger indicated by the greater number of spiral terraces, compared to the features in Fig. 4a, b. This result indicates a change of growth mode from 2 to 3D island with the increase in the film thickness from 2 to 24 nm.

## Conclusion

In this work, we have systematically studied the growth, structure, and morphology of the Cr_1+*δ*_Te_2_ films epitaxially grown on mica flexible substrates. The main findings are: (1) XRD results show a structural change of the films from multiple phases to a single phase with the increase in growth temperature. (2) XPS spectra reflect a desorbed phenomenon of the deposited Te atoms during the growth process. (3) SEM images display an irregular hexagonal-mesh-structure surface morphology of the films, which has not been previously reported in the literature. (4) STM images show that the films would undergo a growth-mode change from 2D at the initial stage in small thicknesses, to a transition growth mode between 2 and 3D at the middle stage in intermediate thicknesses, and to 3D islands at the later stage in large thicknesses, and reveal that the mesh structure consists of nano-islands with the bending surface is induced by the screw dislocations. As such, Cr_1+*δ*_Te_2_ family materials provide a platform for further studies on the fundamental research of vdW heterostructures formed by non-vdW materials and layered substrates. Our work is also expected to facilitate the development of 2D vdW magnetic materials in flexible electronics and spintronics device applications.

## Supplementary Information

**Additional file 1. Figure A1.** Reflection high energy electron diffraction (RHEED) pattern from the mica substrate after annealing treatment.

**Additional file 2. Figure A2.** TEM image from the 24-nm-thick sample.

**Additional file 3.** Figure A3. XRD results of the films grown at 200 °C, 300 °C, 360 °C, and 460 °C with the Te/Cr flux ratio of 35.

**Additional file 4. Figure A4.** AFM images of the films with the Te/Cr flux ratio of 130 grown at (**a**) 300 °C, (**b**) 360 °C, and (**c**) 460 °C. These figures show a type of hexagonal mesh structure, which is well consistent with SEM images shown in Figures 3(**d**), 3(**f**), and 3(**h**).

**Additional file 5. Table A1.** The stoichiometric ratio between Te and Cr elements of the films with different growth temperatures.

## Data Availability

The datasets used and/or analyzed during the current study are available from the corresponding author on reasonable request.

## Abbreviations

2D: Two-dimensional
TMD: Transition-metal dichalcogenide
AHE: Anomalous Hall effect
THE: Topological Hall effect
vdW: Van der Waals
MBE: Molecular beam epitaxy
XRD: X-ray diffraction
XPS: X-ray photoelectron spectroscopy
SEM: Scanning electron microscopy
STM: Scanning electron microscopy
TEM: Transmission electron microscope
RHEED: Reflection high-energy electron diffraction
LT-STM: Low-temperature scanning electron microscopy
UHV: Ultra-high vacuum
AFM: Atomic force microscope
RMS: Root-mean-square
