# Supplementary material for: Growth, structure, and morphology of van der Waals epitaxy Cr1+δTe2 films
Source: Discov Nano. 2023 Feb 24;18(1):23. doi: 10.1186/s11671-023-03791-y (PMC9958219; doi:10.1186/s11671-023-03791-y)
Supplement: Supplementary file 1 — Additional file 1. Figure A1. Reflection high energy electron diffraction (RHEED) pattern from the mica substrate after annealing treatment. [file 11671_2023_3791_MOESM1_ESM.docx]

**RHEED pattern**


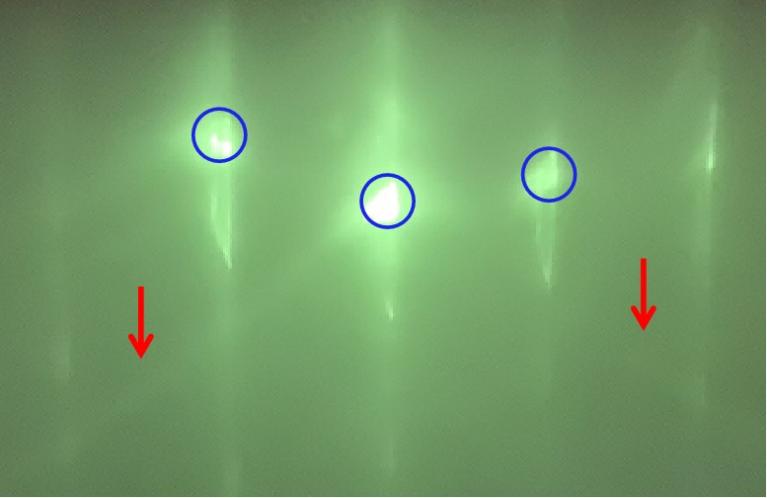


Figure A1. Reflection high energy electron diffraction (RHEED) pattern from the mica substrate after annealing treatment.
