# Supplementary figures and images for: Growth, structure, and morphology of van der Waals epitaxy Cr1+δTe2 films
Source: Discov Nano. 2023 Feb 24;18(1):23. doi: 10.1186/s11671-023-03791-y (PMC9958219; doi:10.1186/s11671-023-03791-y)

**TEM image**


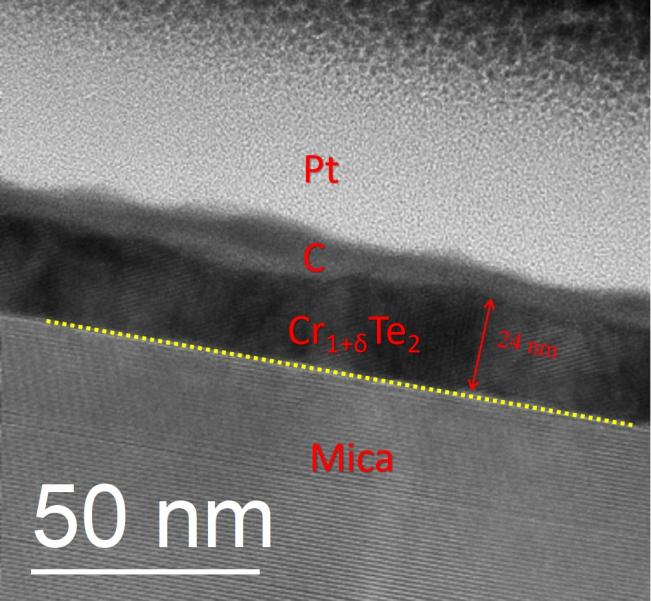


Figure A2. TEM image from the 24-nm-thick sample.

Supplement: Supplementary file 2 — Additional file 2. Figure A2. TEM image from the 24-nm-thick sample. [file 11671_2023_3791_MOESM2_ESM.docx]

**XRD results**


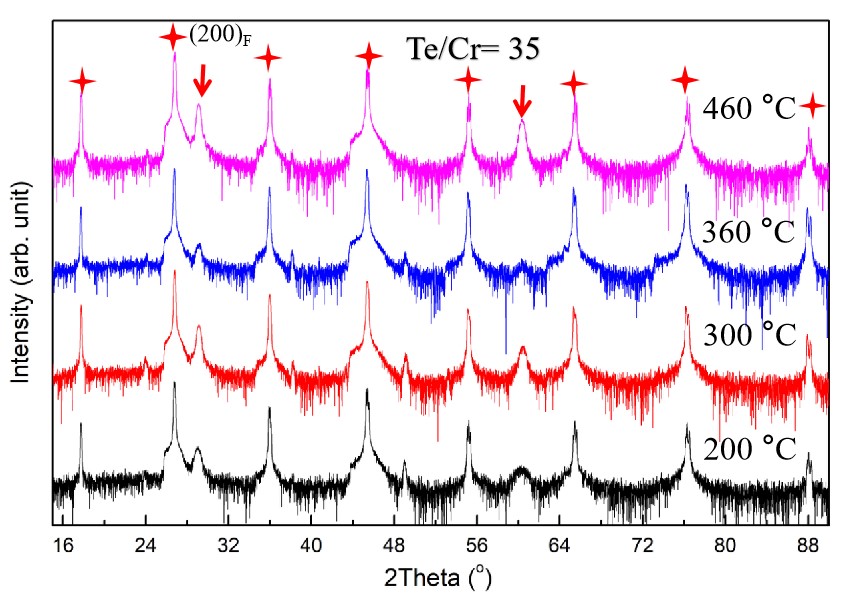


Figure A3. XRD results of the films grown at 200 ℃, 300 ℃, 360 ℃, and 460 ℃ with the Te/Cr flux ratio of 35.

Supplement: Supplementary file 3 — Additional file 3. Figure A3. XRD results of the films grown at 200 °C, 300 °C, 360 °C, and 460 °C with the Te/Cr flux ratio of 35. [file 11671_2023_3791_MOESM3_ESM.docx]
