# Supplementary material for: Growth, structure, and morphology of van der Waals epitaxy Cr1+δTe2 films
Source: Discov Nano. 2023 Feb 24;18(1):23. doi: 10.1186/s11671-023-03791-y (PMC9958219; doi:10.1186/s11671-023-03791-y)
Supplement: Supplementary file 4 — Additional file 4. Figure A4. AFM images of the films with the Te/Cr flux ratio of 130 grown at (a) 300 °C, (b) 360 °C, and (c) 460 °C. These figures show a type of hexagonal mesh structure, which is well consistent with SEM images shown in Figures 3(d), 3(f), and 3(h). [file 11671_2023_3791_MOESM4_ESM.docx]

**AFM images**


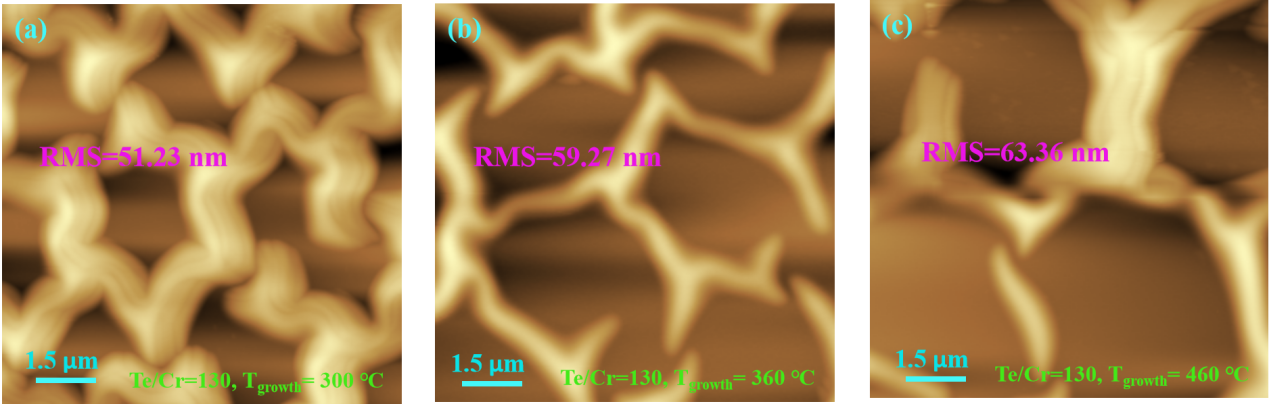


Figure A4. AFM images of the films with the Te/Cr flux ratio of 130 grown at (a) 300 °C, (b) 360 °C, and (c) 460 °C. These figures show a type of hexagonal mesh structure, which is well consistent with SEM images shown in Figures 3(d), 3(f), and 3(h).
