# Supplementary material for: Growth, structure, and morphology of van der Waals epitaxy Cr1+δTe2 films
Source: Discov Nano. 2023 Feb 24;18(1):23. doi: 10.1186/s11671-023-03791-y (PMC9958219; doi:10.1186/s11671-023-03791-y)
Supplement: Supplementary file 5 — Additional file 5. Table A1. The stoichiometric ratio between Te and Cr elements of the films with different growth temperatures. [file 11671_2023_3791_MOESM5_ESM.docx]

**Stoichiometric Te/Cr ratio**

Table A1 lists the results of the stoichiometric ratio between Cr and Te elements according to the fittings of core level spectra, showing that the atomic ratio of Te/Cr is approximately 0.6 for the lower-temperature samples and 0.8 for the higher-temperature samples, respectively. The two atomic ratio values are far away from 100, indicating that the deposited Te atoms are desorbed numerously during the growth process. Besides, the atomic ratio value persisted almost unchanged with the variation of the growth temperature in the higher temperature samples.

**Table A1.** **The stoichiometric ratio between Te and Cr elements of** the films with different growth temperatures.

| Growth  temperature | Normalized intensity | | | | Te/Cr  ratio |
| --- | --- | --- | --- | --- | --- |
|  | Cr 2p_3/2_ | Te 3d _5/2_ | Cr 2p_1/2_ | Te 3d _3/2_ |  |
| 200 ℃ | 11950.89959 | 10540.99086 | 9738.241708 | 7748.877157 | 0.84 |
| 300 ℃ | 12233.18946 | 7992.383756 | 9834.915947 | 6264.083249 | 0.65 |
| 360 ℃ | 12262.59428 | 7623.86802 | 9536.642435 | 5914.560406 | 0.62 |
| 460 ℃ | 12347.72831 | 7966.915736 | 9848.055429 | 6239.453807 | 0.64 |

**Note:** The normalized intensity is determined by $I_{\mathrm{normalized}}= \frac{A}{S}$ ,where A is the peak area estimated from the XPS spectral data, and S is the relative sensitivity factor.
